# Supplementary material for: BrainIAK tutorials: User-friendly learning materials for advanced fMRI analysis
Source: PLoS Comput Biol. 2020 Jan 15;16(1):e1007549. doi: 10.1371/journal.pcbi.1007549 (PMC6961866; doi:10.1371/journal.pcbi.1007549)
Supplement: Attachment — Submitted filename: PCOMPBIOL-D-19-01130.pdf [file pcbi.1007549.s001.pdf]

Manuscript PCOMPBIOL-D-19-01130 presents a collection of interactive tutorials covering the fundamentals of computational neuroimaging through advanced functional MRI analyses. The manuscript is well written and easy to follow. The authors made a brilliant work structuring a well-designed coursework comprising 13 tutorials. There are several options to execute the tutorials online, locally on a desktop/laptop computer or on a High-Performance Computing (HPC) facility, although the tutorials describe only HPC in detail. The materials presented in the results section of the paper show a nice balance between topical depth and engineering standards, which usually compete in this kind of tutorials.

Although I couldn't timely go through all the tutorials in full detail, I have posted in their GitHub repository a list of suggestions/improvements the authors may consider[1] and I will keep reviewing the materials during the upcoming weeks. Besides those minor comments, I would like to bring up some discussion over the following topics:

- License: the GitHub repo does currently not inform about licensing (https://github.com/brainiak/brainiak-tutorials/issues/15). This should be corrected and the manuscript should be updated (data availability section). Correspondingly, all the datasets used throughout the coursework should be listed along with their licenses (maybe just update Table 1 with licenses and cross-reference from the data availability section).
- Please consider redistributing the tutorials via CodeOcean (https://codeocean.com/)
- BIDS - The Brain Imaging Data Structure is not even mentioned. Given that BIDS provides a wealth of educational materials, I believe it would be great to mention it in tutorial 2. This is not to suggest to change all datasets used in the tutorial to BIDS (although their availability in BIDS format would be desirable).
- Most of the tutorials propose exercises - is there a way for students to check their solutions?
- Tutorial 7 was excessively long for me - I would split the HPC section of the end to a separate unit (probably proposed before starting with searchlight).
- The authors explicitly state that "the materials available to learn these methods are limited, **the software is rarely open-source**, and the analyses are often difficult to run on large datasets" in the Author Summary (and then this is suggested at points). I believe that actually, most of the neuroimaging software is open-source. I believe the authors want to refer to reproducibility issues here. The paper would benefit from some discussion about how these tutorials are useful to address the problem of irreproducibility in neuroimaging.
- Limitations: please clearly mention what are the limitations of these tutorials (e.g., no tutorial about GLMs, which is a very standard technique, shallow coverage of containers, etc.). This is not a request for expanding the materials - as I said above they are well designed.

For all these reasons my recommendation is acceptance with minor revisions.

Oscar Esteban
Postdoctoral Fellow, Stanford University

This review is licensed under a Creative Commons Attribution 4.0 International License (CC-BY) - http://creativecommons.org/licenses/by/4.0/.

[1] https://github.com/brainiak/brainiak-tutorials/issues/created_by/oesteban
