# Supplementary material for: BrainIAK tutorials: User-friendly learning materials for advanced fMRI analysis
Source: PLoS Comput Biol. 2020 Jan 15;16(1):e1007549. doi: 10.1371/journal.pcbi.1007549 (PMC6961866; doi:10.1371/journal.pcbi.1007549)
Supplement: Attachment — Submitted filename: review-PCOMPBIOL-D-19-01130.pdf [file pcbi.1007549.s002.pdf]

In this manuscript, Kumar and colleagues describe their recently released BrainIAK tutorials, a collection of resources designed to make MVPA-style analyses accessible to the broader neuroimaging community. As the authors note, there is a relative lack of educational materials for these methods despite their use throughout computational cognitive neuroscience. These tutorials, therefore, are of significant general interest to researchers working in this or related fields. I do, however, have concerns about the presentation of the tutorials in the present manuscript, particularly in their described relationship to previous work.

- The authors' efforts are tremendous and of obvious importance to the field. Nevertheless, by failing to appropriately place this work in the broader landscape of open source, the authors hinder readers from being able to appropriately understand their contributions.

  - The authors fail to mention several prominent open source tool boxes in multiple languages designed to perform related analyses and often accompanied by significant documentation (lines 53, 68-69, 83-84). For example, the Princeton MVPA toolbox (MATLAB), The Decoding Toolbox (MATLAB), pyMVPA (Python), are all omitted from the manuscript. Nilearn (Python) is mentioned as a toolbox mainly for its "data-loading/handling and basic machine learning" capabilities (line 148, 149); however, nilearn also has modules to run e.g., searchlight analysis, described as a "cutting-edge technique" in line 184.

  - The authors note in line 128-129 that "It is a challenge to find training materials on how to run fMRI analyses on a compute cluster." Although providing this information again is undoubtedly valuable, there are in fact training materials available for running fMRI analyses on a compute cluster, particularly from Neurohackademy and NeuroStars. There is also an example slurm submission script for BIDS Apps (Gorgolewski et al., 2017) in a recent preprint from Esteban and colleagues (Esteban et al., 2019).

  - In lines 155-158, the authors explain their choice to make fully pre-processed data (described in Table 1) available alongside the tutorials. This in and of itself is a substantial contribution, for which the authors should be applauded. However, I have two concerns with the data release as described at present.

    The first is that as with the tutorials themselves (see below), it is unclear under which license these datasets are being released. This is important if readers wish to publish on the datasets in their own work. Could the authors please list the appropriate license for each of these datasets, perhaps in Table 1 ?

    The second concern is that it would be helpful if the authors could reference broader data-sharing initiatives in neuroimaging for readers to discover other (optionally preprocessed) publicly available datasets through platforms such as OpenNeuro. This would empower a novice reader to perform novel analyses with the covered techniques, beyond just those datasets directly linked in the tutorials.

It is also unclear why the data downloads on the website link to google drive, when several of the datasets such as Sherlock and Raider are available from open source repositories with better long-term archiving.

- The tutorials themselves should be more prominently linked throughout the manuscript, particularly in the abstract and in the *Tutorial Notebooks* section (line 259) -- neither of which, at present, contain any direct link to the online materials.

- The tutorials are also referred to throughout the manuscript as "released publicly and freely" (lines 57, 186); however, the licensing of these tutorials is not directly stated either in the manuscript or on the website, where the reader is instead directed to the GitHub repository of the BrainIAK toolbox itself. Given the existence of multiple open source licenses, the authors should instead state the exact license. Inferring from the GitHub repository, I believe this is Apache 2.0.

Minor concerns:

- The authors note that they have developed "user friendly tutorials… and exercises" (line 36-37); however, the difference between these two types of learning materials is never explained, and the exercises are not referenced again. Please clarify what is intended, here.

- The authors note that the "most powerful analyses are complex and computationally intensive" (line 51-52). This is a subjective statement and depends entirely on the research question at hand.

- In the *Other resources* section (line 341) the interested reader is directed to an online GitHub file where resources related to git, python, cluster computing, machine learning, and related topics have been collated. This is a valuable reference, but there are also several other larger, community-sourced collections of similar material, such as from the recent OHBM 2019 Hackathon. It would be valuable to link interested readers to these sources as well.

References
Esteban, O. et al. (2019). Analysis of task-based functional MRI data preprocessed with fMRIPrep. *BioRxiv*. doi: 10.1101/694364
Gorgolewski, K. J. et al. (2017). BIDS apps: Improving ease of use, accessibility, and reproducibility of neuroimaging data analysis methods. PLOS Computational Biology, 13, e1005209. doi: 10.1371/journal.pcbi.1005209
