# Supplementary material for: BrainIAK tutorials: User-friendly learning materials for advanced fMRI analysis
Source: PLoS Comput Biol. 2020 Jan 15;16(1):e1007549. doi: 10.1371/journal.pcbi.1007549 (PMC6961866; doi:10.1371/journal.pcbi.1007549)
Supplement: Attachment — Submitted filename: Brainiak Tutorials Paper_response_letter_v3.pdf [file pcbi.1007549.s003.pdf]

Brainiak Tutorials Paper: Revision

Dear Dr. Marinazzo,

Thank you for giving us the opportunity to undertake a revision of our manuscript, titled "BrainIAK tutorials: user-friendly learning materials for advanced fMRI analysis" (PCOMPBIOL-D-19-01130).

We would like to thank the reviewers for their comments, which have helped improve this manuscript. Below we list each reviewer's comments point-by-point, along with our replies and the changes that were made to the manuscript. Our responses are indented with respect to the reviewers' comments and modified text from the manuscript is placed in quotes.

We have also enhanced our tutorials based on suggestions on GitHub. The NeuroLibre team provided detailed reviews on the first three notebooks, and we incorporated those suggestions and updated those notebooks on NeuroLibre. We have also addressed 9 suggestions raised on GitHub by Dr. Oscar Esteban.

We hope that you will find this revision appropriate and the manuscript suitable for publication in *PLOS Computational Biology*.

Reviewer 1

Manuscript PCOMPBIOL-D-19-01130 presents a collection of interactive tutorials covering the fundamentals of computational neuroimaging through advanced functional MRI analyses. The manuscript is well written and easy to follow. The authors made a brilliant work structuring a well-designed coursework comprising 13 tutorials. There are several options to execute the tutorials online, locally on a desktop/laptop computer or on a High-Performance Computing (HPC) facility, although the tutorials describe only HPC in detail. The materials presented in the results section of the paper show a nice balance between topical depth and engineering standards, which usually compete in this kind of tutorials.

Although I couldn't timely go through all the tutorials in full detail, I have posted in their GitHub repository a list of suggestions/improvements the authors may consider and I will keep reviewing the materials during the upcoming weeks. Besides those minor comments, I would like to bring up some discussion over the following topics:

> We are grateful for your detailed and supportive comments. We have been working during the revision process to update the tutorials based on your helpful suggestions on GitHub. Just prior to submitting this revision, we verified that all of the pending suggestions had been resolved. We will continue to address any issues that arise pre and post publication.

- *License: the GitHub repo does currently not inform about licensing (https://github.com/brainiak/brainiak-tutorials/issues/15). This should be corrected and the manuscript should be updated (data availability section). Correspondingly, all the datasets used throughout the coursework should be listed along with their licenses (maybe just update Table 1 with licenses and cross-reference from the data availability section).*

Thank you for pointing this out. We have updated the license for the tutorials on GitHub to Apache 2.0. We have also licensed the datasets under the Creative Commons Attribution 4.0 International License and updated Table 1 accordingly.

- *Please consider redistributing the tutorials via CodeOcean (https://codeocean.com/)*

We are currently in the process of distributing these tutorials via NeuroLibre (https://conp-pcno.github.io), a free platform to run and execute the tutorials. No installation is required by the user. This platform is user-friendly and is widely available to the general public, so we believe that it achieves the same goal as distributing via CodeOcean. Having said that, we are big fans of CodeOcean and – going forward – we plan to work with them and other platforms to increase the reach of the tutorials.

- *BIDS - The Brain Imaging Data Structure is not even mentioned. Given that BIDS provides a wealth of educational materials, I believe it would be great to mention it in tutorial 2. This is not to suggest to change all datasets used in the tutorial to BIDS (although their availability in BIDS format would be desirable).*

Thank you for this suggestion. We are increasingly using BIDS in other work and agree that it is important to highlight the general issue of file naming and header conventions. We now highlight this issue in Notebook 2 and we provide a link to the BIDS educational materials. BIDS data are compatible with our tutorials, as the tutorials can easily be modified to suit any directory and file naming structure. We have provided an example in Notebook 2 about how to read data files in BIDS format.

- *Most of the tutorials propose exercises - is there a way for students to check their solutions?*

As these materials were designed for classroom teaching -- and we hope others will adopt them for the same purpose -- we intentionally left the answers out. However, we do have a solution key that we will finalize after the review process to incorporate any revisions to the notebooks. The solution key will be hosted as a private repo and will be provided to users upon request if they affirm that they are not currently students in a for-credit course using these materials. We have also added a note to the public repo that instructors interested in using the tutorials in courses are encouraged to contact the creators for additional information (at which point the solutions will be shared, along with other classroom advice based on three semesters of teaching).

- *Tutorial 7 was excessively long for me - I would split the HPC section of the end to a separate unit (probably proposed before starting with searchlight).*

We have found that the best way to teach the use of HPC is by linking it to a neuroscientific goal (here, searchlight analysis). A standalone HPC notebook would be different in character from all of the other surrounding notebooks, which are based on fMRI methods rather than generic computing topics. For these reasons, we prefer to keep Tutorial 7 as a single tutorial. Having said this, we understand the reviewer's concern; to address this concern, we have added a message at

the start of the HPC section in the notebook mentioning that this will take a long time to execute in non-HPC environments. This will allow users to plan their effort in completing the notebook, and also allow users on clusters to get the full benefit of the tutorials.

● *The authors explicitly state that "the materials available to learn these methods are limited, **the software is rarely open-source**, and the analyses are often difficult to run on large datasets" in the Author Summary (and then this is suggested at points). I believe that actually, most of the neuroimaging software is open-source. I believe the authors want to refer to reproducibility issues here. The paper would benefit from some discussion about how these tutorials are useful to address the problem of irreproducibility in neuroimaging.*

In the revision, we have highlighted the problem of reproducibility in psychology and neuroscience (see below for changes). The tutorials are inherently designed to reproduce findings with open data and open code. We are hopeful this will encourage further transparency when users adapt the tutorial code for their research studies.

To address the reviewer's concerns listed above, we have added the following text:

p. 4, lines 53-55:
"Furthermore, the materials available to learn these methods do not encompass all the methods used, work is often published with no publicly available code, and the analyses are often difficult to run on large datasets without cluster computing."

p. 6 lines 88-90:
"One barrier to increasing the accessibility of these techniques is that, in most cases, they were created as custom code within individual labs and are thus not part of other fMRI software analysis packages."

p. 11 lines 203-208:
"We have released these tutorials publicly and freely. The users can also apply these methods to publicly available datasets from the existing literature, leading to independent validation of the published results. We are hopeful that this will help increase reproducibility of results more broadly: when tutorial users analyze their own data, they will have already become familiar with the tools necessary to share their code and data, leading to a cycle of improved data sharing and code validation."

Lastly, we agree that most neuroimaging software is open source these days, however, some of packages require paid licenses (e.g. Princeton MVPA Toolbox, through Matlab). We have added the following text to the Introduction (p. 5-6, lines 83-88):

"There exist multiple open-source packages that implement MVPA techniques and RSA. Some of these packages require paid MATLAB licenses (e.g. Princeton MVPA Toolbox, The Decoding Toolbox [17], and CoSMoMVPA [18]) and others are completely free (e.g. Nilearn [19]and PyMVPA [20,21]). Although all these packages cover a broad range

of MVPA and RSA techniques, they do not cover techniques such as FCMA, ISC, ISFC, SRM, and event segmentation."

- *Limitations: please clearly mention what are the limitations of these tutorials (e.g., no tutorial about GLMs, which is a very standard technique, shallow coverage of containers, etc.). This is not a request for expanding the materials - as I said above they are well designed.*

Our goal in these tutorials was to focus on advanced fMRI analysis techniques, and hence we consciously decided not to cover GLMs or preprocessing, or to spend much time on the deployment components such as containers themselves. We agree that these are gaps and limitations, which we have now outlined in the paper, directing readers to other resources (p. 20, lines 382-386):

"Furthermore, our goal for these tutorials was to cover advanced fMRI analysis and hence our tutorials do not cover pre-processing methods, General Linear Model analysis, or software deployment options (e.g., containers) in great detail. An exhaustive list covering multiple helpful tools and tutorials is available here: https://github.com/ohbm/hackathon2019/blob/master/Tutorial_Resources.md."

For all these reasons my recommendation is acceptance with minor revisions.

Oscar Esteban
Postdoctoral Fellow, Stanford University

Thank you again for these detailed and constructive suggestions, which we believe have strengthened the paper and tutorials.

Reviewer 2

In this manuscript, Kumar and colleagues describe their recently released BrainIAK tutorials, a collection of resources designed to make MVPA-style analyses accessible to the broader neuroimaging community. As the authors note, there is a relative lack of educational materials for these methods despite their use throughout computational cognitive neuroscience. These tutorials, therefore, are of significant general interest to researchers working in this or related fields. I do, however, have concerns about the presentation of the tutorials in the present manuscript, particularly in their described relationship to previous work.

Thank you for your detailed and helpful comments. As described below, we have modified the introduction to position our work in the context of other available open-source packages.

- *The authors' efforts are tremendous and of obvious importance to the field. Nevertheless, by failing to appropriately place this work in the broader landscape of open source, the authors hinder readers from being able to appropriately understand their contributions.*
    - *The authors fail to mention several prominent open source tool boxes in multiple languages designed to perform related analyses and often accompanied by significant documentation (lines 53, 68-69, 83-84). For example, the Princeton MVPA toolbox (MATLAB), The Decoding Toolbox (MATLAB), pyMVPA (Python), are all omitted from the manuscript. Nilearn (Python) is mentioned as a toolbox mainly for its "data-loading/handling and basic machine learning" capabilities (line 148, 149); however, nilearn also has modules to run e.g., searchlight analysis, described as a "cutting-edge technique" in line 184.*

We regret this significant oversight. Our intention was not to diminish these important contributions and we completely agree that highlighting them and their relationship is important.

The Matlab packages had been excluded simply because we were focusing on packages that did not require paid software licenses. In retrospect, mentioning them serves to acknowledge the historical context and to highlight the relevance of the current work to users of those packages. As creators of the Princeton MVPA Toolbox, we view BrainIAK as the next iteration of that project, bringing it into an open-source framework, with considerably expanded functionality, and with more professional coding, documentation, and high-performance capabilities.

pyMVPA and Nilearn are directly relevant and deserve a more thorough treatment. We have now discussed these packages in the Introduction (p. 5-6, lines 83-95). Nilearn provides an interface with algorithms relevant to the contents of some of the tutorials, namely classification, regression, feature selection, dimensionality reduction, and searchlight. BrainIAK creates an expressive environment in which these and several other cutting-edge methods have been implemented, including ISC, ISFC, SRM, FCMA, TFA, Bayesian RSA. The tutorials highlight many of these functions. We are hopeful that researchers will expand and adapt the tutorials to leverage more functions from BrainIAK and other packages. To reflect these points, we have made the following changes:

p. 5, lines 83-88:

"There exist multiple open-source packages that implement MVPA techniques and RSA. Some of these packages require paid MATLAB licenses (e.g. Princeton MVPA Toolbox, The Decoding Toolbox [17], and CoSMoMVPA [18]) and others are completely free (e.g. Nilearn [19] and PyMVPA [20,21]). Although all these packages cover a broad range of MVPA and RSA techniques, they do not cover techniques such as FCMA, ISC, ISFC, SRM, and event segmentation."

p. 6, lines 93-95:
"The tutorials also show how to use methods in other packages such as Nilearn and how to integrate them with the methods in BrainIAK."

p. 9, lines 164-166:

"For data loading we use Nibabel[27]; for data masking, normalization, dimensionality reduction, plotting, atlases, and functional connectivity we use Nilearn[19]; and for machine learning libraries we use Scikit-learn[28]."

We have also corrected the statement about searchlights (p. 7, lines 126-129): "For many of the cutting-edge techniques in BrainIAK, no tutorials exist (one notable exception is the volumetric searchlight technique; tutorials for this method are included in the PyMVPA [20,21] and Nilearn [19] packages) or they are taught only as a part of special workshops."

○ *The authors note in line 128-129 that "It is a challenge to find training materials on how to run fMRI analyses on a compute cluster." Although providing this information again is undoubtedly valuable, there are in fact training materials available for running fMRI analyses on a compute cluster, particularly from Neurohackademyand NeuroStars. There is also an example slurm submission script for BIDS Apps (Gorgolewski et al., 2017) in a recent preprint from Esteban and colleagues (Esteban et al., 2019).*

Thank you for bringing these helpful materials to our attention, which we have now referenced in the paper (p. 8 , lines 138-142).

"It is a challenge to find training materials on how to run fMRI analyses on a compute cluster, although, resources are becoming increasingly available, for example, lectures on Neurohackademy (https://neurohackademy.org/course_type/lectures/); and forums such as NeuroStars (https://neurostars.org) for using fmriprep on clusters [25]."

○ *In lines 155-158, the authors explain their choice to make fully pre-processed data (described in Table 1) available alongside the tutorials. This in and of itself is a substantial contribution, for which the authors should be applauded.*

Thank you.

However, I have two concerns with the data release as described at present.
The first is that as with the tutorials themselves (see below), it is unclear under which license these datasets are being released. This is important if readers wish to publish on the datasets in their own work. Could the authors please list the appropriate license for each of these datasets, perhaps in Table 1 ?

This was an inadvertent omission on our part. We have licensed the datasets under the Creative Commons Attribution 4.0 International License and updated Table 1 (p. 12) accordingly.

The second concern is that it would be helpful if the authors could reference broader data-sharing initiatives in neuroimaging for readers to discover other (optionally preprocessed) publicly available datasets through platforms such as OpenNeuro. This would empower a novice reader to perform novel analyses with the covered techniques, beyond just those datasets directly linked in the tutorials.

Great idea! We use and contribute to OpenNeuro regularly, and agree that this is a fantastic resource for expanded use of the tutorial code. We now highlight resources with publicly available datasets for this purpose (p. 15 lines 283-286):

"The user is also encouraged to make novel contributions using the method that they learned in the tutorial, either by enhancing the method, creating a new visualization of the data, or even using the method on another dataset, e.g., from OpenNeuro (http://openneuro.org)."

It is also unclear why the data downloads on the website link to google drive, when several of the datasets such as Sherlock and Raider are available from open source repositories with better long-term archiving.

In testing prior to release, we discovered that the fastest download speeds were achieved using Google Drive and hence delivered these datasets using Google Drive. We also provide ready-to-use masks and smaller extracts of the datasets, which can save time (especially on Google Colaboratory) for the novice user and enable execution on platforms with limited resources. The tutorials website (brainiak.org/tutorials) also links to a Zenodo version of the same datasets, which serves as a long-term archive.

That said, to avoid any confusion, we also now list (p. 12, Table 1) for each dataset other repositories in which it is available (if any), and highlight what is different about the version we provide (e.g., preprocessing, masks, etc.).

- *The tutorials themselves should be more prominently linked throughout the manuscript, particularly in the abstract and in the Tutorial Notebooks section (line 259) -- neither of which, at present, contain any direct link to the online materials.*

We have added links to the tutorials page in the Abstract and the Tutorial Notebooks section, as suggested

p. 2, lines 36-37:

"To disseminate these powerful tools, we developed user-friendly tutorials (in Jupyter format; https://brainiak.org/tutorials/)"

p. 16, line 294-295:

"We describe the contents of each notebook (https://brainiak.org/tutorials) in more detail below:"

- *The tutorials are also referred to throughout the manuscript as "released publicly and freely" (lines 57, 186); however, the licensing of these tutorials is not directly stated either in the manuscript or on the website, where the reader is instead directed to the GitHub repository of the BrainIAK toolbox itself. Given the existence of multiple open source licenses, the authors should instead state the exact license. Inferring from the GitHub repository, I believe this is Apache 2.0.*

Good point, we have updated the license for the tutorials to Apache 2.0 on GitHub.

Minor concerns:

- *The authors note that they have developed "user friendly tutorials... and exercises" (line 36-37); however, the difference between these two types of learning materials is never explained, and the exercises are not referenced again. Please clarify what is intended, here.*

Thank you for pointing this out. We will clarify this. The exercises are a part of the tutorials and may be used to learn the materials and/or may be used as part of a formal course assignment. We have made the following changes:

p. 9 lines 156-161 now read as follows:

"For all users, we embed background material and references, prompts for further self-study, and problem set exercises to help them learn how to generate and adapt code. The exercises for each notebook focus on neuroscientific applications of the techniques being learned; thus, by working through the exercises, students learn how to use these techniques to answer meaningful neuroscientific questions (course instructors may contact us for more information)."

p. 15 lines 277-281 now read as follows:

"The accompanying notebook exercises help the user understand the method and its applicability to the scientific question by requiring that they generate answers or code. These questions are posed in the context of a publicly available fMRI dataset. These questions and exercises can be used to formally evaluate students enrolled in a for-credit course (course instructors may contact us for more information)."

- *The authors note that the "most powerful analyses are complex and computationally intensive" (line 51-52). This is a subjective statement and depends entirely on the research question at hand.*

We have modified the Author Summary and clarified this with the following statement (p. 4, lines 51-53):

"However, exhaustive multivariate analyses in space and time, run across a large number of subjects, can be complex and computationally intensive, creating a high barrier for entry into this field."

- *In the Other resources section (line 341) the interested reader is directed to an online GitHub file where resources related to git, python, cluster computing, machine learning, and related topics have been collated. This is a valuable reference, but there are also several other larger, community-sourced collections of similar material, such as from the recent OHBM 2019 Hackathon. It would be valuable to link interested readers to these sources as well.*

We agree and have added a link to the OHBM 2019 hackathon resource page when we reference "Other Resources" (p. 20, lines 384-386):

"An exhaustive list of helpful tools and tutorials is available here: https://github.com/ohbm/hackathon2019/blob/master/Tutorial_Resources.md"

Reviewer 3

Reviewer #3: # Summary and general comments

In this submission, Kumar and colleagues present a library called BrainIAK for machine learning in functional neuroimaging, and an accompanying set of tutorials.
The tutorials are presented in the form of jupyter notebooks, and are accessible either locally through containers or online on the google collab platform.
They also include instructions for deployment on high-performance infrastructure.
The data used in the tutorial are freely available and specially prepared to be used as part of a training activity.
As a strength, some of the material covered in the tutorials include inter-subject correlations and representational similarity analysis, two applications which are not well covered by currently available tutorials, to my knowledge.
Overall, this new library and tutorials are remarkably comprehensive, and I believe will represent a very valuable resource for the community. My only major concern is that the authors did not properly position their work compared to other efforts.

Thank you for these positive impressions and constructive comments. We have modified the Introduction to position our work in the context of other available packages, which we agree was missing before and is important for advancing the field collaboratively (p. 5-6, lines 83-88):

"There exist multiple open-source packages that implement MVPA techniques and RSA. Some of these packages require paid MATLAB licenses (e.g. Princeton MVPA Toolbox, The Decoding Toolbox [17], and CoSMoMVPA [18]) and others are completely free (e.g. Nilearn [19]and PyMVPA [20,21]). Although all these packages cover a broad range of MVPA and RSA techniques, they do not cover techniques such as FCMA, ISC, ISFC, SRM, and event segmentation."

# Minor comments
** abstract: citing a specific list training and hackathons will become obsolete in a few months only. Maybe stay vague there.*

We have edited out these references from the abstract (p. 2, lines 43-45):

"These notebooks were successfully tested at multiple sites, including as problem sets for courses at Yale and Princeton universities and at various workshops and hackathons."

** intro claims several times the lack of existing education material. There is a huge amount of general-purpose tutorials for machine learning, most notably featuring the sklearn documentation.*

We agree completely and have benefited tremendously from such materials. Our focus was exclusively on machine learning in the context of neuroimaging and so we did not emphasize general-purpose tutorials, such as from sklearn. In the tutorials we do occasionally provide links to specific aspects of sklearn and nilearn for additional help. We further added a link to the sklearn learning materials in the Introduction when we discuss general-purpose machine learning,

and tailored our claims about the lack of education materials to be specifically about machine learning in neuroimaging.

p. 7, lines 121-126 are now modified as follows:
"There are multiple tutorials on machine learning available (see examples on Scikit-learn https://scikit-learn.org/stable/auto_examples/index.html); however, only a few cover the use of machine learning in cognitive neuroscience: for example, the documentation for Nilearn[21], lectures from the MIND summer school, lectures from the Organization for Human Brain Mapping education section and hackathons, and blogs such as MVPA Meanderings."

There are at least three extensive packages with many tutorials: nilearn, pyMVPA and https://www.ncbi.nlm.nih.gov/pmc/articles/PMC4956688/. The authors should briefly review these other resources and explain how BrainIAK adds to them (paragraph line 120). Which techniques are not currently covered by tutorials?

We have elaborated on the contribution of BrainIAK and its tutorials with respect to the other packages in more detail in the following paragraphs:

In the Author Summary we have removed the statement that materials are unavailable and the modified lines are as follows (p. 4, lines 53-55):
"Furthermore, the materials available to learn these methods do not encompass all the methods used, work is often published with no publicly available code, and the analyses are often difficult to run on large datasets without cluster computing."

In the Introduction we added (p. 5-6, lines 83-88):
"There exist multiple open-source packages that implement MVPA techniques and RSA. Some of these packages require paid MATLAB licenses (e.g. Princeton MVPA Toolbox, The Decoding Toolbox [17], and CoSMoMVPA [18]) and others are completely free (e.g. Nilearn [19]and PyMVPA [20,21]). Although all these packages cover a broad range of MVPA and RSA techniques, they do not cover techniques such as FCMA, ISC, ISFC, SRM, and event segmentation."

p. 6, lines 93-95:
"The tutorials also show how to use methods in other packages such as Nilearn and integrate them with the methods in BrainIAK."

We list the limitations of the tutorials (p. 20, lines 382-386):
"Furthermore, our goal for these tutorials was to cover advanced fMRI analysis and hence our tutorials do not cover pre-processing methods, General Linear Model analysis, or software deployment options (e.g., containers) in great detail. An exhaustive list covering multiple helpful tools and tutorials is available here: https://github.com/ohbm/hackathon2019/blob/master/Tutorial_Resources.md."

We further list techniques that exist in BrainIAK but that are not currently covered by the tutorials in the Future Work section (p. 20, lines 390-392):
"The methods/tools that are available in BrainIAK but not covered in the tutorials are: Bayesian derived methods for RSA; Topographic Factor Analysis; and an fMRI Simulator."

** l. 211: would you have recommendations for resources to preprocess the data that would integrate well with BrainIAK? In particular, you may want to discuss if detailed instructions are available for importing minimally preprocessed data, such as the ones generated by fMRIprep.*

We have added examples of preprocessing pipelines that a user could use. We have added a description in Notebook 2 on how to access data in BIDS format by showing users how to form the required file name string with a task name, space name, and run id. This will allow them to read data files in BIDS format. We have also added a link to additional resources in the "Other Resources" section.

p. 13, lines 238-244:
"The user is free to use any preprocessing pipeline (e.g., fmriprep, AFNI). Data are exchanged in standard NIFTI and NumPy formats with existing tools such as Nibabel or Nilearn and our tutorials show how to import data into Python structures and use BrainIAK. Data are exchanged in standard NIFTI and NumPy formats with existing tools such as Nibabel or Nilearn and our tutorials show how to import data into Python structures and use BrainIAK. The functions in BrainIAK parse the data in a time x voxels format, with an exception being the searchlight function that takes in 4-D volumes. The BrainIAK package also serves as an ecosystem for users to contribute their own methods while avoiding duplication of methods found in other packages."

p. 20, lines 384-386:
"An exhaustive list covering multiple helpful tools and tutorials is available here: https://github.com/ohbm/hackathon2019/blob/master/Tutorial_Resources.md."

** no material is presented to demonstrate that the proposed material achieves the stated goals. Survey results from a workshop, for example, would add some support to the usefulness of the resources.*

From informal discussions at hackathons, workshops, and on our Gitter channel, participants have told us that the tutorials have helped them tremendously. We also have formal evaluations from students who have used these materials in courses at Yale and Princeton and they have been overwhelmingly positive (e.g., the most recent incarnation received a course rating of 4.8/5); we don't think that these institutional evaluations can be made publicly available. We are aware of two final projects from this course that are now in preparation for journal submission. Within our labs, these tutorials are now the starting point for all new lab members. We have also distributed them widely to colleagues, who have expressed gratitude and also use them for graduate training. We realize that this feedback is anecdotal. We will create a survey page on the BrainIAK tutorials

homepage when this paper is published for people to provide feedback and suggestions, which could be posted publicly with consent.

# Optional suggestions

Below are two suggestions. I (as a reviewer) do not think it is necessary to implement these suggestions prior to publication. I am providing these suggestions in the hope the authors may find them useful and may choose to follow up on some of them.
** BrainIAK should go through a proper code review, as a library. Consider a submission to the journal of open source software (JOSS) for the library component of BrainIAK.*

Thank you for this helpful suggestion, JOSS seems ideal for this purpose.

** I have not reviewed the tutorials themselves, but tried to evaluate if BrainIAK adds conceptually to existing software resources. As part of the NeuroLibre platform, a detailed technical review of the notebooks has been performed by two reviewers. I would encourage the authors to address these technical issues.*

Thanks to Prof. Bellec and the NeuroLibre team for a detailed review of each notebook (so far Notebooks 1-3 have been reviewed). We have already resolved all comments for Notebooks 1-3 and the pull requests have been merged into the NeuroLibre GitHub repository. As the other notebooks are reviewed, we will work towards incorporating them as well.
