# Supplementary material for: BrainIAK tutorials: User-friendly learning materials for advanced fMRI analysis
Source: PLoS Comput Biol. 2020 Jan 15;16(1):e1007549. doi: 10.1371/journal.pcbi.1007549 (PMC6961866; doi:10.1371/journal.pcbi.1007549)
Supplement: Attachment — Submitted filename: PCOMPBIOL-D-19-01130_R1.pdf [file pcbi.1007549.s004.pdf]

The revision of manuscript PCOMPBIOL-D-19-01130 successfully addressed all my comments. Based on that, my recommendation is to accept the paper.

That said, I disagree with some of the edits the authors introduced to address my comments about how this paper would help address the problems of reproducibility (or lack thereof) in the field. I would like to invite the authors to reconsider carefully some of these edits (it might be that we just have different opinions - if that is the case, then the authors should not modify their work based on this feedback).

In p. 11, lines 203-208, the authors introduced the following changes:

> "*We have released these tutorials publicly and freely. The users can also apply these methods to publicly available datasets from the existing literature, leading to independent validation of the published results. We are hopeful that this will help increase reproducibility of results more broadly: when tutorial users analyze their own data, they will have already become familiar with the tools necessary to share their code and data, leading to a cycle of improved data sharing and code validation.*"

The authors argue that ensuring neuroimagers are knowledgeable enough to execute existing methods is fundamental to run replication attempts of existing literature (which I agree). However, I disagree that will help increase reproducibility - I believe it will actually weigh in the opposite direction: we will discover more and more studies that don't replicate. This is because there's no interest in using outdated methods (and methodologies that have been proved wrong) in data analysis. This is not a pessimistic view of reality, it is just that many findings will be proven an artifact of faulty analyses and poorly understood statistics (which is great in conjunction with the self-correcting property of Science). What I'm trying to argue is that improving the formation of new scientists will help the reproducibility of Science onwards, but not backwards. I'm also arguing that investigating which findings in the literature are not replicable is important, but does not contribute to improving the overall reproducibility on the field.

> Lastly, we agree that most neuroimaging software is open source these days, however, some of packages require paid licenses (e.g. Princeton MVPA Toolbox, through Matlab). We have added the following text to the Introduction (p. 5-6, lines 83-88):
> "*There exist multiple open-source packages that implement MVPA techniques and RSA. Some of these packages require paid MATLAB licenses (e.g. Princeton MVPA Toolbox, The Decoding Toolbox [17], and CoSMoMVPA [18]) and others are completely free (e.g. Nilearn [19]and PyMVPA [20,21]). Although all these packages cover a broad rangeof MVPA and RSA techniques, they do not cover techniques such as FCMA, ISC, ISFC, SRM, and event segmentation.*"

I think the authors confuse Open-Source with Licensing. Software can simultaneously be commercial and open-source. Science is more reproducible when the instruments we use are not black-boxes (i.e., in terms of reproducibility, it is important for the software to perform transparently and that is achieved by making the source code available). Commercial software, when open-source, just limits scientists in accessing software but does not preempt them to check the code (i.e., the software can still operate transparently).

As the authors will see, these two comments can be viewed as academic nitpicking. However, some of these statements could be a result of trying to address referees' comments in a timely manner, at the cost of precision. I believe they have a wonderful opportunity to provide the community with a thoughtful view on

these matters. I invite them to reconsider these positions carefully, although the paper is ready for publication from the referee's standpoint.

Oscar Esteban
Postdoctoral Fellow, Stanford University

This review is licensed under a Creative Commons Attribution 4.0 International License (CC-BY) - http://creativecommons.org/licenses/by/4.0/.
